# Taxonomy of the African large carpenter bees of the genus *Xylocopa* Latreille, 1802, subgenus *Xenoxylocopa* Hurd & Moure, 1963 (Hymenoptera, Apidae)

**DOI:** 10.3897/zookeys.655.11187

**Published:** 2017-01-13

**Authors:** Jonathan R. Mawdsley

**Affiliations:** 1 Department of Entomology, MRC 187, National Museum of Natural History, Smithsonian Institution, P. O. Box 37012, Washington, DC 20013-7012 USA

**Keywords:** Africa, Apidae, Hymenoptera, identification, Large carpenter bee, pollinator, taxonomy, *Xylocopa*

## Abstract

The taxonomy of the genus Xylocopa Latreille, 1802, subgenus Xenoxylocopa Hurd & Moure, 1963, is reviewed. There is a single valid species in this subgenus, Xylocopa (Xenoxylocopa) inconstans Smith, 1874, which is widely distributed throughout sub-Saharan Africa, from Senegal to Ethiopia and south to northern Republic of South Africa. Synonyms of *Xylocopa inconstans* include *Xylocopa abyssinica* Radoszkowski, 1899, proposed for a male specimen from Ethiopia, as well as three names proposed for females with yellow (rather than white) dorsal pubescence: *Mesotrichia chiyakensis* Cockerell, 1908 (new synonym), Xylocopa inconstans var. flavescens Vachal, 1899, and Xylocopa inconstans var. flavocincta Friese, 1909. Quantitative analyses of body measurements and examination of male reproductive structures support the new synonymy of *Mesotrichia chiyakensis* with *Xylocopa inconstans*. Males and females of Xylocopa (Xenoxylocopa) inconstans are illustrated, along with male reproductive structures, and diagnostic characters and keys are provided to separate the males and females of Xylocopa (Xenoxylocopa) inconstans from those of species in other closely-allied African subgenera of the genus *Xylocopa*.

## Introduction

Large carpenter bees, species of the genus *Xylocopa* Latreille, 1802, are important floral visitors and pollinators of flowering plants in many terrestrial ecosystems, including both agricultural and non-agricultural settings (Hurd and Moure 1963; Gerling et al. 1989; Keasar 2010; Mawdsley et al. 2016). The genus *Xylocopa* has its greatest diversity in the tropics, with over 700 species currently recognized in the genus (Hurd and Moure 1963; Hurd 1978; Michener 2007). Certain tropical subgenera within the genus *Xylocopa* are poorly known from a taxonomic viewpoint and in need of revision (Hurd and Moure 1963; Michener 2007). This paper reviews the taxonomic history of a single subgenus, Xylocopa (Xenoxylocopa) Hurd & Moure, 1963, which occurs throughout much of sub-Saharan Africa.

## Taxonomic review

Hurd and Moure (1963: 243–247) created the subgenus Xenoxylocopa for the reception of five names proposed by earlier authors for African species in the genus *Xylocopa* Latreille, 1802 (Latreille 1802: 379): *Xylocopa inconstans* Smith, 1874 (Smith 1874: 264), *Xylocopa abyssinica* Radoszkowski, 1899 (Radoszkowski 1899: 127), Xylocopa inconstans var. flavescens Vachal, 1899 (Vachal 1899: 146), Xylocopa inconstans var. flavocincta Friese, 1909 (Friese 1909: 253), and *Mesotrichia chiyakensis* Cockerell, 1908 (Cockerell 1908: 34). The type species of the subgenus Xenoxylocopa was fixed by original designation by Hurd and Moure (1963: 243) as *Mesotrichia chiyakensis* Cockerell.

As noted by Eardley (1983), the taxonomy of species in this subgenus has been based largely on characters of body size and coloration, particularly the color of the pale pubescence on the mesosoma and metasoma of the females, which ranges from white to bright yellow. The first of the five names in this group to be published was *Xylocopa inconstans* Smith, 1874, which was based on an unspecified number of representatives of the female sex from South Africa and from Lake Ngami in present-day Botswana (Eardley 1983). Smith (1874) noted that the specimens available to him for study exhibited considerable variation in size and in coloration, with body length ranging from “ten to twelve lines” (approximately 21 to 30 mm) and the color of the lighter pubescence ranging from “snow white” to “bright yellow.” Radoszkowski (1899) extended the range of *Xylocopa inconstans* northward to Ethiopia, based on examination of an unspecified number of white and black female specimens, and proposed the name *Xylocopa abyssinica* for a male specimen from Ethiopia. Vachal (1899) described Xylocopa inconstans var. flavescens for specimens from Senegal with yellow setae on the scutellum. Cockerell (1908) described *Mesotrichia chiyakensis* based on a large-bodied female specimen from “Chiyaka, Benguella, West Africa” (in modern-day Angola) with yellow pubescence on the thorax and abdomen, and provided a key to separate females from those of *Xylocopa inconstans* and *Xylocopa flavescens*, which he treated as full species in the genus *Mesotrichia* Westwood, 1838. Friese (1909) reviewed the names published to date in this group, treating *Xylocopa abyssinica* and *Xylocopa chiyakensis* as synonyms of *Xylocopa inconstans* and describing a variety Xylocopa inconstans var. flavocincta for female specimens from multiple localities (Gambia, Togo, Madibura and Kwidjwi on Lake Kivu, and Cheren in Eritrea) with yellow pubescence on the scutellum and abdomen. LeVeque (1928) discussed the taxonomic placement and morphological characters of species in this group, transferred its species from *Mesotrichia* to *Xylocopa* on the basis of adult morphological characters, and illustrated the male reproductive structures of a specimen of *Xylocopa chiyakensis* which had been collected by members of the American Museum of Natural History’s Congo Expedition (Osborn 1919) in what is now the Democratic Republic of the Congo.

The taxonomy of the southern African members of this subgenus was reviewed by Eardley (1983), who placed or confirmed placement of three of the five names proposed in this subgenus (*Xylocopa abyssinica*, Xylocopa inconstans var. flavescens, and Xylocopa inconstans var. flavocincta) as synonyms of a single variable species, Xylocopa (Xenoxylocopa) inconstans, based on his study of primary type specimens. Eardley (1983) provided a redescription and illustrations of *Xylocopa inconstans*, including illustrations of male reproductive structures, mapped the distribution of this species in southern Africa, and provided keys and diagnostic characters for separating adults of both sexes of *Xylocopa inconstans* from the other southern African species of the genus *Xylocopa*.

Following the work of Eardley (1983), only two species names are recognized in Xylocopa subgenus Xenoxylocopa: Xylocopa (Xenoxylocopa) inconstans and Xylocopa (Xenoxylocopa) chiyakensis. The most recent subgeneric classification of the genus *Xylocopa*, that of Michener (2007), treated *Xenoxylocopa* as a valid subgenus within *Xylocopa*. Michener (2007) also provided keys for the separation of representatives of the subgenus Xenoxylocopa from those of other subgenera in the genus *Xylocopa*.

## Material examined

The author recently had the opportunity to study the large collection of African carpenter bees in the U. S. National Museum of Natural History (USNM), assembled by the late P. Hurd. This collection includes a small series (2 males, 4 females) of Xylocopa (Xenoxylocopa) chiyakensis which had been collected during the Congo Expedition of the American Museum of Natural History. These specimens formed part of the larger series of specimens that had originally been studied by LeVeque (1928) in the only significant study of *Xylocopa chiyakensis* following its description, and all specimens bore LeVeque’s handwritten identification labels. This series included the male specimen and accompanying reproductive structures which had been dissected and illustrated by LeVeque (1928).

The USNM material also included 41 specimens of Xylocopa (Xenoxylocopa) inconstans collected at the following localities throughout the species’ range: Botswana: Kasane; Democratic Republic of the Congo: Faradje, Garamba, Katanga, Murowe, Park Upemba; Ethiopia: Awasa, Jimma, Lanyani, Melka; Kenya: Stony Athi; Malawi: Southeast shore of Lake Malawi, between Fort Maguire and Fort Johnston; Mozambique: Massangena; Namibia: Shamvura, Kavango Province; Niger: 26 miles W Tapoa; Nigeria: Olokenji, Ibadan; Republic of South Africa: Kruger National Park, Skukuza; Tanzania: Iringa, Mbeya, Nandete; Zambia: Mbala; Zimbabwe: Harare.

To test the assertion of Cockerell (1908) that female specimens with yellow pubescence have larger body size than females with white pubescence, a set of basic body measurements (head capsule width, total body length, and right forewing length) were recorded for all female specimens examined. Average values of these measurements were then calculated separately for female specimens with yellow dorsal pubescence and for female specimens with white dorsal pubescence.

Male reproductive structures from specimens collected in association with females of each color form (yellow and white) were also examined, including the dissected structures which had been illustrated by LeVeque (1928).

## Results

### Status of Xylocopa (Xenoxylocopa) chiyakensis

As noted above, Eardley (1983) reviewed the taxonomic status of all names included in Xylocopa subgenus Xenoxylocopa by Hurd and Moure (1963) except *Xylocopa chiyakensis*, which was described from material collected in Angola outside the geographic scope of Eardley’s study. The name *Xylocopa chiyakensis* (Cockerell, 1908) has been applied by authors including LeVeque (1928) and Hurd and Moure (1963) to large-bodied female carpenter bees belonging to Xylocopa subgenus Xenoxylocopa which have yellow (as opposed to white) pubescence on the sides of the mesosoma, the scutellum, and the base of the metasoma. However, the material of this subgenus in USNM also includes both small-bodied females with yellow pubescence and large-bodied females with white pubescence, suggesting that the color characters provided by Cockerell (1908) may not actually correlate with the body size characters.

Cockerell (1908) used total body length and forewing length as diagnostic characters in a key to separate his *Mesotrichia chiyakensis* (said to have total body length 30 mm and forewing length 26 mm) from *Xylocopa inconstans* (said to have total body length of 26 mm and forewing length 21–23 mm). In the material examined, females with yellow pubescence (n = 7) had an average head capsule width of 9.1 mm (range 8.2–10.0 mm) while females with white pubescence (n = 17) had an average head capsule width of 9.0 mm (range 8.0–10.2 mm). Females with yellow pubescence had an average total body length of 27.6 mm (range 24.5–30.0 mm) while females with white pubescence had an average total body length of 25.3 mm (range 19.8–29.0 mm). Females with yellow dorsal pubescence had an average right forewing length of 24.7 mm (range 21.8–30.0 mm) while females with white dorsal pubescence had an average right forewing length of 24.0 mm (range 21.0–30.0 mm).

Given the significant overlap in these sets of measurements between female specimens with yellow pubescence and female specimens with white pubescence, and the fact that the averages of these measurements differ by just 0.1 mm (average head capsule width), 2.3 mm (average total body length), and 0.7 mm (average right forewing length), it does not appear that female specimens with yellow pubescence and female specimens with white pubescence differ significantly in these measurements of body size. Thus, the use of female body size to separate *Xylocopa chiyakensis* and *Xylocopa inconstans*, as proposed in the key of Cockerell (1908), does not appear to be tenable.

Male reproductive structures of *Xylocopa chiyakensis* were illustrated by LeVeque (1928) and those of *Xylocopa inconstans* were illustrated by Eardley (1983). Given the significant differences in size and style of these authors’ illustrations, it is difficult to tell from a comparison of these illustrations alone whether significant diagnostic differences are actually present. For this study, I examined the original dissection prepared by LeVeque (1928) from which her line drawing of the male genitalia of *Xylocopa chiyakensis* was derived. From my examination of this dissection, it is clear that the illustration provided by LeVeque (1928) shows the male genital capsule rotated slightly, suggesting that the dissected parts were likely drawn with a camera lucida or similar device, and thus the outline and general appearance of these structures may not be strictly comparable to the illustrations provided by Eardley (1983). Furthermore, the broad lines and small overall size of the illustration provided by LeVeque (1928) actually obscure important structural features of the genital capsule. Accordingly, I have provided a new, photographic image of the dorsal and ventral surfaces of this dissected male genital capsule here (Figure 4). Comparison with my own dissections of *Xylocopa inconstans* male reproductive structures from other localities and comparison with the illustrations provided by Eardley (1983) of the male reproductive structures in *Xylocopa inconstans* do not reveal significant differences between male reproductive structures in these two forms.

LeVeque (1928) mentioned that the male specimens available to her of *Xylocopa chiyakensis* from central Africa had slightly darker and more brownish pubescence than the male of *Xylocopa inconstans*, which she knew only from the description by Radoszkowski (1876). Differences in coloration can provide important diagnostic information at the species level for males of *Xylocopa* species (see, for example, Eardley 1983 and Hurd 1978). However, the male specimens that I examined from LeVeque’s material had evidently been immersed in some preservative prior to pinning, as their pubescence is matted and discolored. This may be the source of the reported color variation, as the male specimens that I examined from northern Zambia and southwestern Tanzania all had lighter colored pubescence which is similar in color to that of males from southern Africa and Ethiopia.

Finally, Eardley (1983) presents compelling evidence that females with yellow pubescence and females with white pubescence are both present in southern African populations of *Xylocopa inconstans*, and that in at least one instance females of the two different color forms were even found within the same nest.

Given this combined evidence, and the lack of clear, reliable diagnostic features which could be used to separate *Xylocopa chiyakensis* and *Xylocopa inconstans*, I have no difficulties in placing *Xylocopa chiyakensis* in synonymy with *Xylocopa inconstans*, new synonymy. Given the fact that the original description of *Xylocopa inconstans* by Smith (1874) emphasized both the body size variation and the color variation that occurs among females of the species, it is frankly difficult to understand how and why the yellow female color form was subsequently given three separate Latin names by three different workers.

The full synonymy for this species is therefore as follows:

*Xylocopa inconstans* Smith, 1874

*Xylocopa abyssinica* Radoszkowski, 1876

Xylocopa inconstans var. flavescens Vachal, 1899

*Mesotrichia chiyakensis* Cockerell, 1908, new synonym

Xylocopa inconstans var. flavocincta Friese, 1909

### Separation of species of the subgenus Xenoxylocopa from those of other African subgenera of the genus *Xylocopa*

Females and males of the subgenus Xenoxylocopa can be readily separated from species in the other African subgenera of the genus *Xylocopa* using combinations of adult external morphological characters. Females and males of the widespread subgenus Koptortosoma Gribodo, 1894 are most similar to the species of subgenus Xenoxylocopa in terms of their overall appearance and coloration in both males and females (Hurd and Moure 1963). In my experience and that of my colleagues (Mawdsley et al. 2016), certain common and widespread species of the subgenus Koptortosoma are often found in association with the species of the subgenus Xenoxylocopa; however, species of these two subgenera can be readily separated using certain combinations of adult external morphological characters. In particular, the following characters will help to separate species of *Xenoxylocopa* from sympatric species in the closely allied subgenera *Koptortosoma* and *Mesotrichia* Westwood, 1838.

**Figure 1–4.**
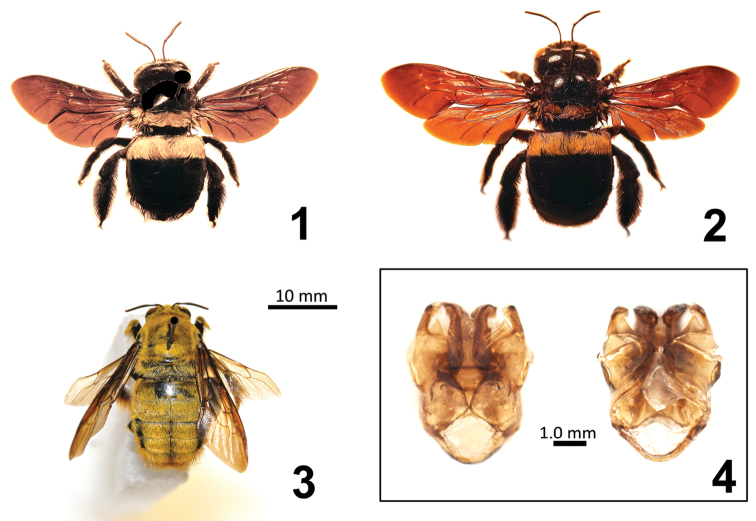
**1** Adult female of Xylocopa (Xenoxylocopa) inconstans Smith, collected by the author at Skukuza, Kruger National Park, Mpumalanga, Republic of South Africa, on flowers of *Peltophorum africanum* Sonder (Fabaceae) **2** Adult female of Xylocopa (Xenoxylocopa) inconstans Smith, collected at Garamba, Democratic Republic of the Congo, and identified by Norma LeVeque as *Xylocopa chiyakensis* (Cockerell) **3** Adult male of Xylocopa (Xenoxylocopa) inconstans Smith, collected at Kruger National Park, Mpumalanga, Republic of South Africa **4** Dorsal (left) and ventral (right) views of the male genital capsule of Xylocopa (Xenoxylocopa) inconstans Smith, collected at Faradje, Democratic Republic of the Congo, dissected and illustrated by Norma LeVeque (1928, figure 16) as the male genitalia of *Xylocopa chiyakensis* (Cockerell).

*Female* (Figures 1, 2). Head capsule broad and massive, space behind eyes greatly expanded. The space behind eyes is not greatly expanded and the head capsule is not as broad and massive in females of subgenera *Koptortosoma* and *Mesotrichia*.

First metasomal tergite lacking a distinct invaginated acarinarium or “mite chamber.” The acarinarium is present in females of subgenera *Koptortosoma* and *Mesotrichia*.

Small tooth present on either side of the median pygidial spine. This tooth is absent in females of subgenera *Koptortosoma* and *Mesotrichia*.

*Male* (Figures 3, 4). Frons very broad, maximum distance between compound eyes across frons greater than the vertical length of the compound eyes. The frons is narrower in males of subgenera *Koptortosoma* and *Mesotrichia*.

Inner margin of compound eyes with a distinct groove parallel to and adjacent to the eye margin. This groove is absent in males of subgenera *Koptortosoma* and *Mesotrichia*.

### Key to subgenera of *Xylocopa* in the “Mesotrichia group” from Continental Africa

Michener (2007) provides keys for separating species of subgenus Xenoxylocopa from the other Old World subgenera of *Xylocopa*, while Eardley (1983) provides keys for separation of both sexes of *Xylocopa inconstans* from the other species of *Xylocopa* in southern Africa. At this writing, the Michener (2007) keys and text are available online at: http://base.dnsgb.com.ua/files/book/Agriculture/Beekeeping/Thep-Bees-of-the-World.pdf.

The following keys were developed to separate species of the subgenus Xenoxylocopa from species of continental African subgenera which belong to what I call here the “*Mesotrichia* Group,” a morphologically well-defined group of Old World subgenera within *Xylocopa* which may ultimately prove to be a distinct monophyletic group (as suggested by the phylogenetic analyses of Minckley (1998)). Females of the continental African species in this group can be readily separated from those of other continental African *Xylocopa* subgenera by the presence of a sharp transverse ridge on the scutellum which divides the scutellum into two distinct surfaces, a more or less horizontal dorsal anterior surface and a more or less vertical posterior surface. Males of the continental African species in this group of subgenera have a somewhat similar modification to the first metasomal tergite, which is divided by a transverse ridge into a more or less horizontal posterior surface and a more or less vertical or sloping anterior surface. In other continental African subgenera of *Xylocopa*, the scutellum is rounded or feebly angled in females, while the first metasomal tergite is sloping or rounded in males. The following key is based on adult morphological characters which were originally identified and used in keys by Hurd and Moure (1963), Eardley (1983), and Michener (2007), and which were confirmed through my own examination of specimens of these subgenera in USNM.

**Table:** 

1	Antenna with 10 flagellomeres, metatibiae with two tibial spurs, sting apparatus present, pygidial plate present, females	**2**
–	Antenna with 11 flagellomeres, metatibiae with one tibial spur, sting apparatus absent, pygidial plate absent, males	**4**
2	First metasomal tergite lacking a distinct invaginated acarinarium or “mite chamber,” head capsule greatly enlarged and massive, pygidium with a small tooth on either side of the median pygidial spine	**Xylocopa (Xenoxylocopa)**
–	First metasomal tergite with distinct invaginated acarinarium or “mite chamber,” head capsule not greatly enlarged, pygidium lacking small tooth on either side of the median pygidial spine	**3**
3	Elevated ridge of scutellum extending beyond posterior margin of metanotum	**Xylocopa (Koptortosoma)**
–	Elevated ridge of scutellum not extending beyond posterior margin of metanotum	**Xylocopa (Mesotrichia)**
4	Mesothoracic legs strongly modified, with spines and/or flattened areas	**Xylocopa (Mesotrichia)**
–	Mesothoracic legs not strongly modified	**5**
5	Frons very broad, maximum distance between compound eyes across frons greater than the vertical length of the compound eyes; inner margin of compound eyes with a distinct groove parallel to and adjacent to the eye margin	**Xylocopa (Xenoxylocopa)**
–	Frons narrower, maximum distance between compound eyes across frons less than the vertical length of the compound eyes; inner margin of compound eyes lacking a distinct groove parallel to and adjacent to the eye margin	**Xylocopa (Koptortosoma)**
